# Telomere length variation does not correspond with the growth disturbances in the rainbow trout (*Oncorhynchus mykiss*)

**DOI:** 10.1007/s13353-021-00669-6

**Published:** 2021-11-13

**Authors:** Ligia Panasiak, Karolina Szubert, Marcin Polonis, Konrad Ocalewicz

**Affiliations:** 1 Department of Marine Biology and Ecology, Institute of Oceanography, University of Gdansk, M. Piłsudskiego 46 Av, 81378 Gdynia, Poland; 2 Department of Marine Biotechnology, Institute of Oceanography, University of Gdansk, M. Piłsudskiego 46 Av, 81-378 Gdynia, Poland

**Keywords:** Body size, Dwarfism, Growth, Telomerase, Telomeres

## Abstract

Somatic growth is considered to affect pace of the telomere attrition in vertebrates. As normally developed and dwarf fish differ in the body size we have decided to compare telomere length in the rainbow trout (*Oncorhynchus mykiss*) with normal growth and with growth reduced due to the dwarf condition. Examined 1-year-old fish with normal and dwarf appearance were siblings originated from androgenetic fully homozygous doubled haploid (DH) line of rainbow trout. Particular dwarf individuals had body deformities such as humpback, kyphosis, and lordosis. Somatic cells of examined rainbow trout had an average telomere length between 17 and 20 kb, comparable in females and males. Dwarf rainbow trout exhibited significantly lower body length and weight than their normally developed siblings even though no differences in the telomere length were found between these fishes. Statistical analysis did not exhibit any correlation between body size and the telomere length. Equal length of telomeres observed in the studied normal and dwarf rainbow trout suggests morphological and physiological differences in fish with different growth rates do not affect dynamics of telomeric DNA. Or any variation in the telomere length might have been levelled by telomerase that in rainbow trout is active in all tissues irrespective of the individual developmental stage.

**Supplementary Information:**

The online version contains supplementary material available at 10.1007/s13353-021-00669-6.

## Introduction

Telomeres, the nucleoprotein complexes located at the ends of linear eukaryotic chromosomes that assure their complete replication, protect their ends from DNA degradation, DNA repair mechanism, and fusions with other chromosomes (De Lange 2002; Chan and Blacburn 2004; Bolzán and Bianchi, 2006). Telomeres shorten with every cell division as the DNA polymerase is not able to replicate ends of the linear chromosomes (“end replication problem”) (Olovnikov 1973). Furthermore, shortening of telomeres is accelerated by the oxidative stress associated with increased production of reactive oxygen species (ROS) (Von Zglinicki 2002). ROS are mainly produced in mitochondria during process of oxidative metabolism, thus somatic growth as energetically costly process is considered to affect dynamics of telomere attrition (Von Zglinicki 2002). Somatic growth may lead to decrease telomere length by two main paths; by increased cell division rate that is indispensable for growing of the organisms (1) or/and by increased energy expenditure and hence increased oxidative stress/ROS generation leading to acceleration of DNA damage (Monaghan and Ozanne 2018). Shortening of telomeres can be slowed by telomerase that catalytic component, TERT (telomerase reverse transcriptase), adds telomeric DNA repeats using as a template an integral RNA element (TR, telomerase RNA). Telomerase activity in humans is restricted to germ line cells, stem cells, and tumors (Chan and Blackburn 2004). In turn, in fish, activity of telomerase has been confirmed in many organs irrespective of size and age of examined specimens [Klapper et al. 1998; Elmore et al. 2008; Lund et al. 2009].

Scoliosis, lordosis, or kyphosis are quite frequently observed in fish from the wild stocks as well as from the aquaculture (Boglione et al. 2013). Due to the increased metabolic costs, somatic growth in individuals with spinal deformities may be retarded what usually results in dwarfism (Witten et al. 2009; McMenamin et al. 2013; Shao et al. 2019). In experiments concerning production of rainbow trout (*Oncorhynchus mykiss*) androgenetic and gynogenetic doubled haploids (DHs), dwarf individuals with spinal deformities have been frequently observed (Ocalewicz et al. 2010, 2013, among others). Growth retardation makes dwarf fish interesting objects to study dynamics of the telomeric DNA. On the one hand, dwarf fish may have decreased ratio of cell divisions; on the other hand, such fish presumably show increased metabolic costs (Shao et al. 2019) what may affect pace of the telomere shortening. Therefore, the main goal of the present research was to examine changes of the telomere length in the dwarf and normal rainbow trout.

## Materials and method

This study was carried out in strict accordance with the recommendations in the Polish ACT of 21 January 2005 of Animal Experiments (Dz. U. of. 2005 No 33, item 289). The protocol was approved by the Local Ethical Committee for the Experiments on Animals in Bydgoszcz.

### Fish origin and breeding conditions

Androgenetic doubled haploids (DHs) and their normal heterozygous siblings studied in this research were produced and maintained using gamete donors from the spring spawning broodstock of rainbow trout from Rutki strain reared in the Department of Salmonid Research (DSR), Inland Fisheries Institute in Olsztyn (IFI), Rutki, Poland (Polonis et al. 2021). Shortly, batches of eggs were divided in to two groups; one designated for irradiation and induction of androgenesis and the second batch to serve as control (normal fertilization). Eggs were irradiated with 350 Gy of X-rays (6 Gy/min) using TrueBeam linear accelerator (Varian Medical Systems, Palo Alto, CA, USA) and two opposed field technique (175 Gy from each field). Eggs from the irradiated and non-irradiated batches were inseminated with 0.15 ml of milt for about 150 g of eggs in the presence of the sperm activating medium (SAM). Eggs were rinsed with the hatchery water 5 min after activation. After 350 min since insemination fertilized and irradiated eggs were exposed to the high hydrostatic pressure (HHP) shock (9500 PSI for 3 min) using TRC‐APV electric/hydraulic apparatus (TRC Hydraulics Inc. Dieppe, Canada), this process is intended to duplicate paternal set of chromosomes and to create androgenetic doubled haploids (DHs). Control groups were provided by fertilization of the non-irradiated eggs from each female. Both androgenetic and control groups were incubated in three replicates at 10 °C under routine hatchery conditions.

### Fish sampling and measurements

One-year-old dwarf androgenetic individuals (*n* = 7), normally developed androgenetic specimens (*n* = 7) and their normally developed siblings from control group (*n* = 7) were sampled and examined. The sample size was determined to meet requirements for the statistical analysis and it was based on the previous studies using Q-FISH performed in zebrafish (Anchelin et al. 2011) and rainbow trout (Panasiak et al. 2020). All rainbow trout were maintained under the same husbandry and environmental conditions. Sampled fish were sacrificed by an overdose of propiscin (Etomidatum, IFI) (Inland Fisheries Institute, Olsztyn, Poland), weighed and their length measured (Table 1).

**Table 1:** Sex, body size, telomere length-related fluorescence (TLF) and estimated telomere length of rainbow trout: androgenetic dwarfs (_d_DH), normal androgenotes (_n_DH), and their siblings from control group (C)

Number of individual	Sex	Body weight [g]	Body length [cm]	TLF	Estimated telomere length [kb]
_d_DH_1_	M	23.56	12	11.95 ± 2.45	16.12
_d_DH_2_	F	45.05	15.9	12.8 ± 5.45	17.27
_d_DH_3_	F	36.07	13.7	15.85 ± 3.7	21.39
_d_DH_4_	M	78.8	16.5	18.4 ± 6	24.83
_d_DH_5_	M	32	15	12.9 ± 4.1	17.41
_d_DH_6_	F	44.1	14.5	11.55 ± 2.1	15.58
_d_DH_7_	M	36.7	14.5	10 ± 1.4	13.49
_n_DH_1_	F	77.8	18.3	13.05 ± 4.1	17.61
_n_DH_2_	M	137.6	23	13.15 ± 3	17.74
_n_DH_3_	F	104.3	21.2	11.25 ± 1.95	15.18
_n_DH_4_	M	143	22	10.6 ± 2.95	14.30
_n_DH_5_	F	148.7	23.5	11.95 ± 1.95	16.12
_n_DH_6_	F	64.5	17.6	13.3 ± 2.25	17.94
_n_DH_7_	F	101.9	21.2	14.35 ± 3.25	19.36
C_1_	M	97.9	21.4	13.15 ± 2.55	17.74
C_2_	F	93.8	20.8	13.55 ± 3.75	18.28
C_3_	M	185.3	24	20.85 ± 4.25	28.13
C_4_	F	86.1	20.5	12.4 ± 2	16.73
C_5_	M	158.8	23	20.25 ± 5.5	27.32
C_6_	F	139.1	23.5	15.25 ± 3.25	20.58
C_7_	F	154.9	23.5	11.55 ± 3.25	15.58

### Preparation of interphase spreads

Somatic cells of all sampled fish were prepared from the head kidney tissue. This particular tissue was chosen, because it is commonly used in the fish cytogenetic analysis for preparation of the interphase spreads (Panasiak et al. 2020). Pieces of the head kidneys were homogenized in 5 ml of KCl (0.075 M) and left for 40 min at room temperature. Afterward, ice cold fixative (methanol: acetic acid, 3:1) was added and the tubes were centrifuged at 1000 rpm for 10 min. Supernatant was removed, fresh fixative was added, and samples were centrifuged again. This step was repeated three times. After last centrifugation, supernatant was replaced by a fresh fixative and cell suspension was transferred to the plastic tubes and stored at − 20 °C.

### Preparation of cell line L5178Y-R

Murine DBA/2 lymphoma cell line L178Y-R (European Collection of Authenticated Cell Cultures via Merck KGaA, Darmstadt, Germany; LOT06/F/004) with known telomere length (79.7 kb) has been used in the present research as a reference for the Q-FISH analysis of the rainbow trout cells and to assess length of the trout telomeric DNA The culture was carried using RPMI1640 medium (Carl Roth GmbH) supplemented with 10% fetal bovine serum (Merck KGaA) and penicillin–streptomycin solution (50u and 0.05 mg per 1 ml of medium respectively; Merck KGaA) and maintained between 3 × 10^4^ to 7 × 10^5^ cells per ml. Incubation was carried out at 37 °C with 5% CO_2_ and continuous shaking at 90 rpm (New Brunswick™ S41i Incubator, Eppendorf AG) in 125-ml single use, vented, PETG Erlenmeyer flasks (Thermo Fisher Scientific Inc.). About 72 h prior to preparation of the interphase spreads, the cells were seeded to 50 ml of fresh culture medium at 10^6^ cells per 1 ml. Incubation was conducted as mentioned above. After 72 h, the whole volume of the culture was centrifuged at 1000 rpm. Interphase spreads were prepared in the same way as the head kidney cells, despite the hypotonization step which lasted 45 min.

### Interphase quantitative fluorescence in situ hybridization (Q-FISH)

Telomeric DNA repeats were detected using a Telomere PNA (peptide nucleic acid) FISH Kit/FITC (DAKO, Glostrup, Denmark) according to the manufacturer’s protocol. Microscopic slides with fish cells and DBA/2 lymphoma cells were prepared the day before PNA-FISH experiment and kept at the room temperature. Q-FISH analysis has been performed using the method described in details in (Panasiak et al. 2020). Shortly, after the PNA-FISH slides were scanned with the ASI system, HiFISH-SpotScan module (Applied Spectral Imaging, Yokne’am Illit, Israel) with a dedicated 5 M CMOS camera connected to a fluorescent BX53 Olympus microscope. Ten fields of view (frames) were captured under 100 × magnification with DAPI and FITC filters in the multiple focal planes to ensure all signals will be in focus for the correct analysis. All cells and the telomere fluorescent signals in the scanned region were automatically detected by HiFISH-SpotScan software. Quantitative analysis of the telomere fluorescence intensity was performed only on the classified cells. Average telomere fluorescence intensities and standard deviations were reported per 100–200 cells from the scanned regions (Supplementary File 1). The intensity values were given in the arbitrary units, as acceptable in such cases.

### Statistical analysis

Provided data were analyzed using R software version 1.3.959 (Supplementary File 2). Normal distribution was tested by Shapiro–Wilk test and Levene’s test was applied to check homogeneity of variances between analyzed groups of rainbow trout. Correlations between telomere length-related fluorescence and fish body length and weight were assessed using Pearson or Spearman test, according to the data distribution. ANOVA was used to determine if there were any significant differences between means of the telomere length-related fluorescence and body size of all the examined groups of fish. Due to lack of variance homogeneity, difference in the body weight between dwarf individuals and fish from control group was estimated using Kruskall-Wallis. *T*-test was applied to determine if there is any significant difference in the telomere length-related fluorescence between normal rainbow trout males (XY) and females (XX) and between androgenetic males (YY) and females (XX).

## Results

**Fig. 1:**
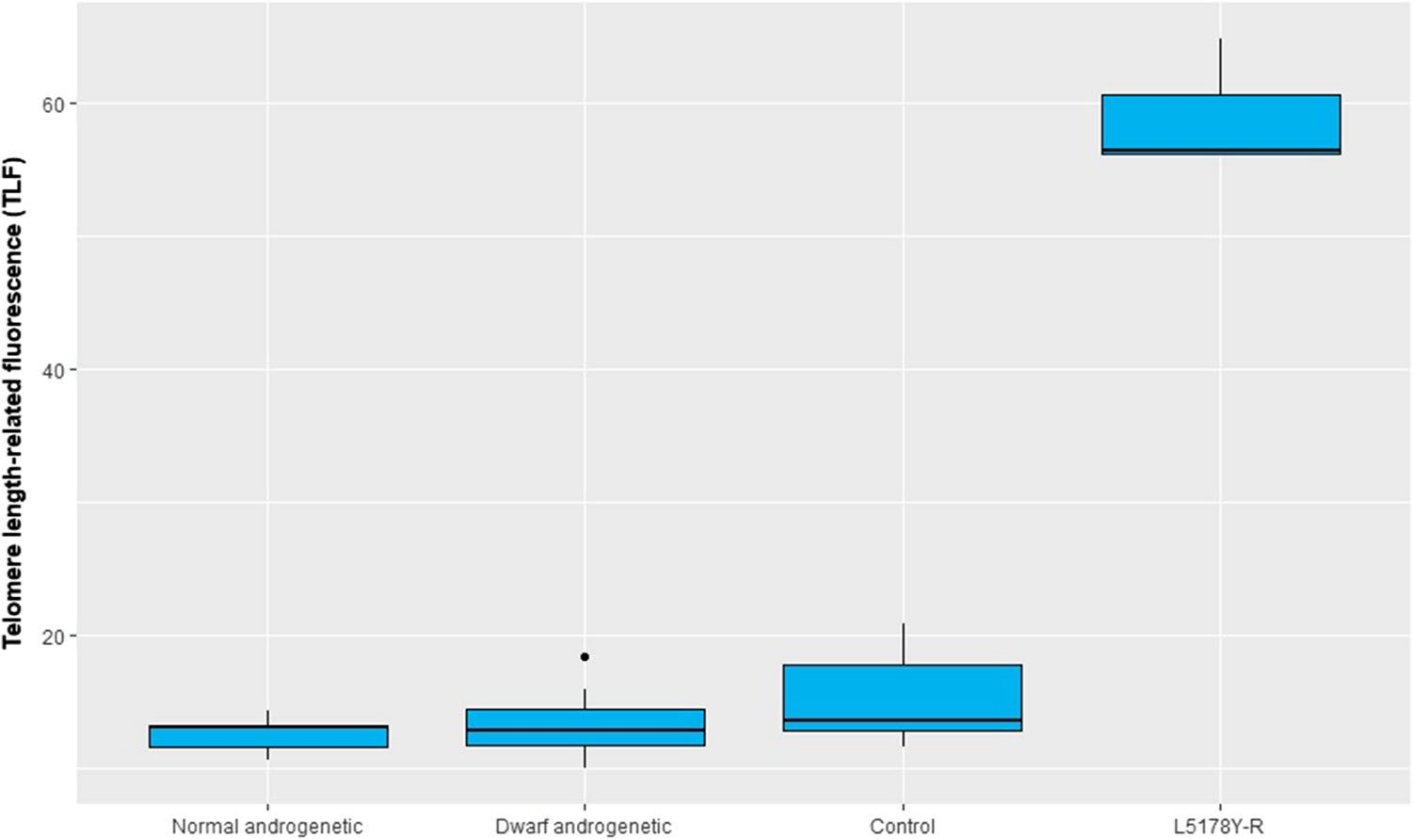
Values of telomere length-related fluorescence (expressed in the fluorescent arbitrary units × 10) in cells from examined rainbow trout and L5178Y-R cells

The mean values of body weight/length of normally developed androgenotes, androgenetic dwarfs`, and heterozygous trout from the control group equaled 111.11 g/20.97 cm, 42.33 g/14.59 cm, and 130.84 g/ 22.39 cm, respectively (Fig. 1, Table 1). Dwarf androgenetic rainbow trout exhibited significantly lower body length and weight when compared to androgenetic fish with normal appearance and trout from the control group (*p* < 0.05). Particular dwarf androgenetic fish had visible morphological changes for example humpback, kyphosis, and lordosis. There were no significant differences in the body size between normally developed androgenetic trout and fish from the control group.

Telomere length-related fluorescence (TLF) (expressed in the fluorescent arbitrary units × 10) in the normally developed androgenetic rainbow trout, androgenetic dwarfs, and control rainbow trout equaled 12.52 ± 2.78, 13.42 ± 3.60, and 15.29 ± 3.51 (mean ± SD), respectively (Supplementary File 1, Fig. 1). For L178Y-R cells, TLF was 59.07 ± 6.93.

**Fig. 2:**
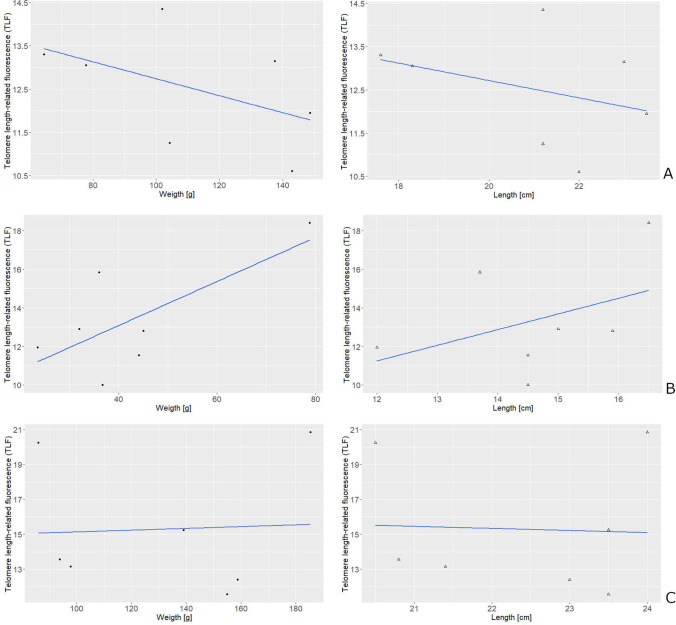
Relationship between telomere length-related fluorescence (TLF) (× 10) and body weight and length in the androgenetic rainbow trout with normal growth rate (*r* = 0.495; *p* = 0.259 and *r* = 0.344; *p* = 0.450, respectively) (**A**), in the androgenetic rainbow trout with growth retardation ( *r* = 0.708; *p* = 0.075 and *r* = 0.421; *p* = 0.347, respectively) (**B**) and in the rainbow trout from control group (*r* = 0.051; *p* = 0.913 and *r* = 0 .458; *p* = 0.922, respectively (**C**)

Results of statistical analysis showed that there were no statistically significant differences between telomere length-related fluorescence in rainbow trout from different examined groups. Statistical analysis did not show any correlation between body weight or length and the telomere length-related fluorescence in the examined fish (Fig. 2). Moreover, no significant differences in the telomere length between females and males were found among androgenetic fish and among their heterozygous siblings from the control group.

## Discussion

A slower growth rate is observed in the large species when compared to the small bodied species; however the opposite is observed within the species where large specimens grow faster than the smaller ones (Monaghan and Ozanne 2018). Due to the increased cell divisions, higher metabolic rate, and related increased generation of ROS, we may expect that fast growing individuals exhibit shorter telomeres. On the other hand, faster growth may be related to better conditions of life and smaller oxidative stress that in turn should slow down pace of the telomere attrition. As normal and dwarf fish differ in the body size and the growth rate, we also expected to see differences in the telomere length between individuals with normal and retarded growth. Average telomere length-related fluorescence (TLF) in 1-year-old rainbow trout was 15.21 (fluorescent arbitrary units × 10) and androgenotes and their heterozygous siblings from the control group had telomeres of similar size. In comparison of TLF in rainbow trout cells and in L5178Y-R cells with known telomere length indicates, that average telomere length of the examined rainbow trout may equal about 20 kb what is in agreement with previously published estimation provided in the course of other than Q-FISH method for the telomere length measurement, namely terminal restriction fragment (TRF) analysis by Southern blot analysis (Lejnine et al. 1995). In spite of significant differences in the body length and weight observed between androgenetic DH rainbow trout with normal growth and those with the retarded growth, no significant differences in the telomere length were found between these fishes (Fig. 2). Provided results have proven that retarded growth observed in the dwarf rainbow trout is not followed by changes in the dynamics of the telomeric DNA. Equal length of telomeres observed in the trout siblings with normal and abnormal body size suggests that any differences in the number and pace of cell divisions, metabolic costs, and thereby oxidative stress in rainbow trout with different growth rate do not affect their telomere length. On the other hand, it is not excluded that any differences in the telomere length resulted from variation in the somatic growth rate and body size between dwarfs and normally developed trout examined here are levelled by telomerase that in rainbow trout is active in all tissues irrespective of individual developmental stage (Klapper et al. 1998).

Except for no differences in the telomere length between normal and dwarf rainbow trout, issue that draws attention was an inter-individual variation of the telomere length in the examined specimens (Table 1). Telomere length is a complex heritable trait and the inter-individual variation in the telomere length appears during early developmental stages as the interaction between genetic and predominant environmental factors including oxidative stress, inflammation, or physiological stress (Srinivas et al. 2020). On the other hand, it is not excluded that such variation was related to the tissue that had been used for cell preparation. Fish head kidney is a hemopoietic organ forming blood elements where processes of hemopoiesis including erythropoiesis, granulopoiesis, monopoiesis, thrombopoiesis, and lymphoplasmopoiesis occurs. The head kidney contains varied types of cells at different stages of maturity (Abdel-Aziz et al. 2010) what may affect telomere length in these cells. Similar inter-individual variation in the telomere length has been confirmed in killifish (*Fundulus heteroclitus*), dogfish shark (*Squalus acanthias*), little skate (*Raja erinacea*), American eel (*Anguilla rostrata*), or zebrafish (Elmore et al. 2008; Lund et al. 2009). Highly heterogeneous telomeres in terms of length have been observed in the liver cells of Japanese medaka (*Oryzias latipes*) (6 to 12 kb) and *O. melastigma* (0.5 to 12 kb) (Au et al. 2009).

In many vertebrate species, females have longer telomeres and live longer than males (Barrett and Richardson 2011). Such pattern of the telomere length and lifespan have been also described in medaka (Gopalakrishnan et al. 2013) and *Nothobranchius furzeri* (Reichard et al. 2020) while in the common carp (*Cyprinus carpio*) (Izzo et al. 2014) and rainbow trout examined here females and males exhibited comparable length of telomeric DNA. Different patterns of telomere dynamics reported between sexes in several fish species may mirror interspecies differences related to the lifespan and sex differentiation. On the other hand, it is not excluded that in the rainbow trout any differences in the telomere length between females and males might appear only in the sexually matured and adult individuals. As oestrogens has been shown to stimulate telomerase activity (Viña et al. 2005), it may be presumed that high level of this steroid observed in females but not in males is at least in part responsible for the lower rates of telomere attrition in females. Studied in the present research, one-year-old rainbow trout were not sexually matured thus inter sex differences in levels of oestrogens might have been too low to affect telomere length in the sex specific manner. To answer these doubts, fully matured rainbow trout females and males should be examined and the sample size needs to be larger.

## Supplementary Information

Below is the link to the electronic supplementary material.

Supplementary file1 (DOC 54 KB)

R-script for conducting analysis related to rainbow trout telomere length and body size. (R 7 KB)
