# Supplementary material for: Telomere length variation does not correspond with the growth disturbances in the rainbow trout (Oncorhynchus mykiss)
Source: J Appl Genet. 2021 Nov 13;63(1):133–9. doi: 10.1007/s13353-021-00669-6 (PMC8755684; doi:10.1007/s13353-021-00669-6)
Supplement: Supplementary file 1 — Supplementary file1 (DOC 54 KB) [file 13353_2021_669_MOESM1_ESM.doc]

|  | TLF | SD |  |  |  | TLF | SD |  |  | TLF | SD |  |  | TLF | SD |
| --- | --- | --- | --- | --- | --- | --- | --- | --- | --- | --- | --- | --- | --- | --- | --- |
| ANDRO1 | 11,7 | 2,5 |  |  | DWARF1 | 11,9 | 2,3 |  | CONTROL1 | 14 | 3,3 |  | CELLS | 64,8 | 8,9 |
| ANDRO2 | 13,5 | 2,7 |  |  | DWARF2 | 12 | 7,9 |  | CONTROL2 | 14,9 | 5,2 |  |  | 56 | 6,1 |
| ANDRO3 | 11,3 | 1,6 |  |  | DWARF3 | 13 | 2,9 |  | CONTROL3 | 23,2 | 4,4 |  |  | 56,4 | 5,8 |
| ANDRO4 | 8,9 | 2,7 |  |  | DWARF4 | 12,6 | 3,1 |  | CONTROL6 | 11,9 | 1,6 |  |  | 59,07 | 6,93 |
| ANDRO5 | 13,9 | 1,5 |  |  | DWARF5 | 11,7 | 1,7 |  | CONTROL5 | 21,7 | 6,1 |  |  |  |  |
| ANDRO6 | 13,7 | 2,5 |  |  | DWARF6 | 10 | 1,6 |  | CONTROL7 | 13,2 | 2,8 |  |  |  |  |
| ANDRO7 | 14,3 | 3,1 |  |  | DWARF7 | 9,4 | 1,4 |  | CONTROL8 | 11,4 | 3,9 |  |  |  |  |
|  |  |  |  |  |  |  |  |  |  |  |  |  |  |  |  |
| ANDRO1 | 14,4 | 5,7 |  |  | DWARF1 | 12 | 2,6 |  | CONTROL1 | 12,3 | 1,8 |  |  |  |  |
| ANDRO2 | 12,8 | 3,3 |  |  | DWARF2 | 13,6 | 3 |  | CONTROL2 | 12,2 | 2,3 |  |  |  |  |
| ANDRO3 | 11,2 | 2,3 |  |  | DWARF3 | 18,7 | 4,5 |  | CONTROL3 | 18,5 | 4,1 |  |  |  |  |
| ANDRO4 | 12,3 | 3,2 |  |  | DWARF4 | 25,2 | 8,9 |  | CONTROL6 | 12,9 | 2,4 |  |  |  |  |
| ANDRO5 | 10 | 2,4 |  |  | DWARF5 | 14,1 | 6,5 |  | CONTROL5 | 18,8 | 4,9 |  |  |  |  |
| ANDRO6 | 12,9 | 2 |  |  | DWARF6 | 13,1 | 2,6 |  | CONTROL7 | 17,3 | 3,7 |  |  |  |  |
| ANDRO7 | 14,4 | 3,4 |  |  | DWARF7 | 10,6 | 1,4 |  | CONTROL8 | 11,7 | 2,6 |  |  |  |  |

**Supplementary File 1.** Results of Q-FISH analysis showing telomere length-related fluorescence (expressed in the fluorescent arbitrary units × 10) in cells from examined rainbow trout.
